# Supplementary material for: Proteomic landscape of imipenem resistance in Pseudomonas aeruginosa: a comparative investigation between clinical and control strains
Source: Front Cell Infect Microbiol. 2025 Oct 16;15:1623154. doi: 10.3389/fcimb.2025.1623154 (PMC12571913; doi:10.3389/fcimb.2025.1623154)
Supplement: Supplementary Figure 1 — Comparative GO term enrichment in P. aeruginosa strains. [file DataSheet1.pdf]

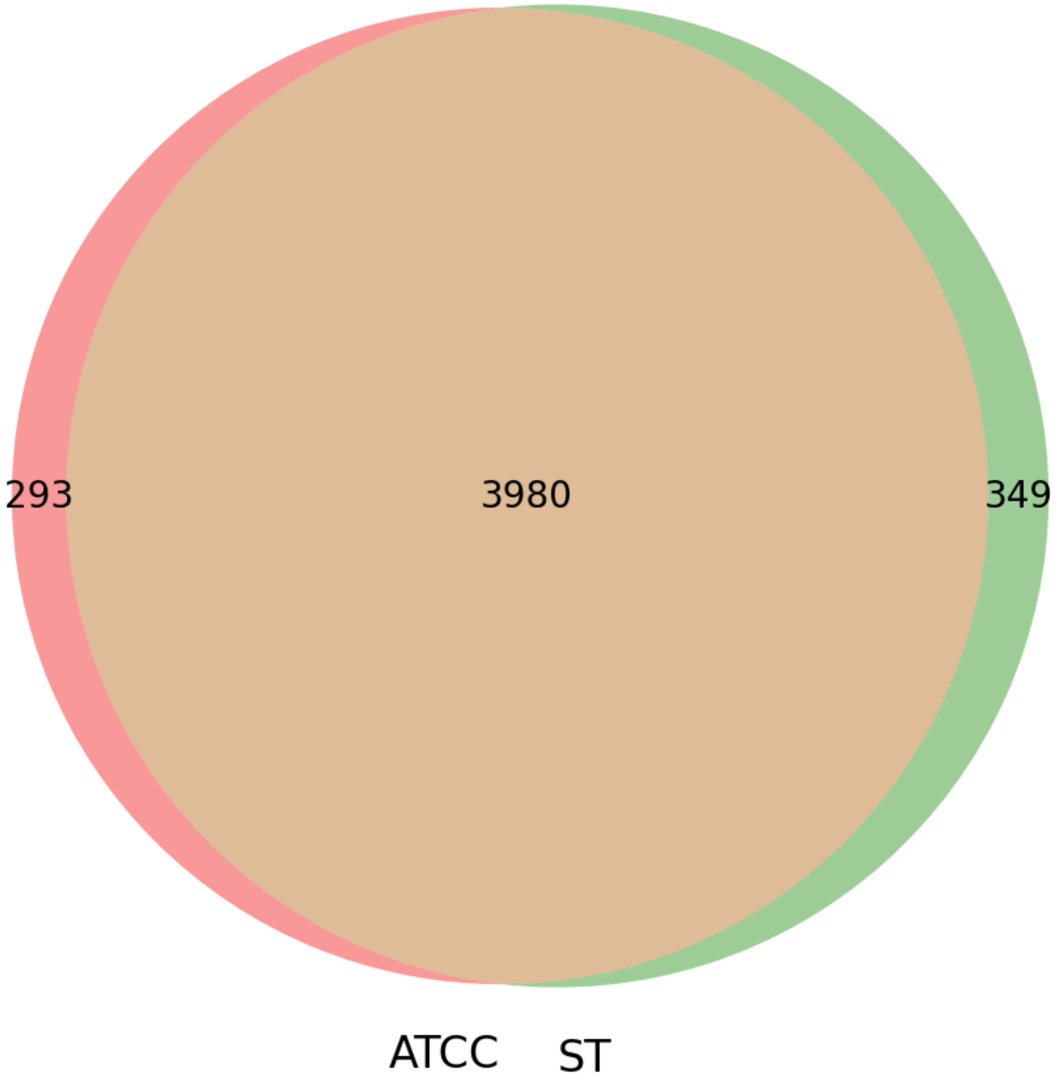

Supplementary Figure S2: Venn diagram showing the number of shared and exclusive genes among control (ATCC 27853 and clinical (ST235) P. aeruginosa strains. *Genomes retrieved from https://www.ncbi.nlm.nih.gov/datasets/genome/GCA_001687285.1/ and https://www.ncbi.nlm.nih.gov/datasets/genome/GCA_016923535.1/ for the control and clinical strains, respectively. BLAST analysis revealed significant hits for the metallo-beta-lactamase (MBL) VIM-2 (https://www.ncbi.nlm.nih.gov/nuccore/GU137304.1) only amongst the unique 349 genes for the clinical strain ST235.*
