# Supplementary material for: Proteomic landscape of imipenem resistance in Pseudomonas aeruginosa: a comparative investigation between clinical and control strains
Source: Front Cell Infect Microbiol. 2025 Oct 16;15:1623154. doi: 10.3389/fcimb.2025.1623154 (PMC12571913; doi:10.3389/fcimb.2025.1623154)
Supplement: Supplementary Figure 2 — Venn diagram showing the number of shared and exclusive genes among clinical and control strains. [file DataSheet2.pdf]

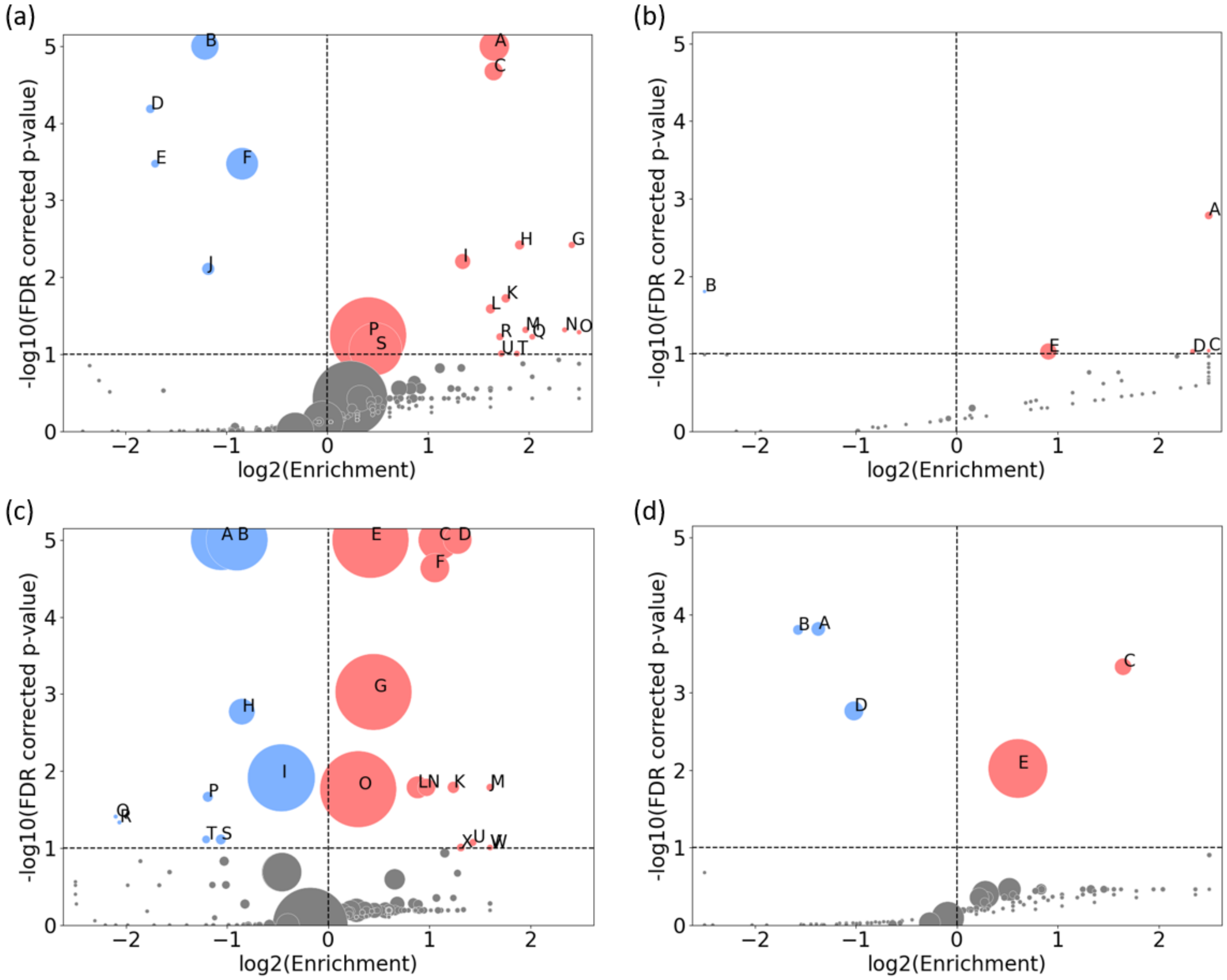

Supplementary Figure S1: Comparative GO term enrichment in P. aeruginosa strains*. The volcano plots depict the enrichment of GO terms in protein subsets from the control strain (a, c) and the clinical strain (b, d), with: (a) a protein-level FDR threshold of 0.1, (b) a protein-level p-value threshold of 0.1 (no FDR as the clinical strain only had 8 proteins adhering to the FDR threshold, which is deemed too small for a sensible GO term analysis for comparative purposes), and (c, d) no p-value threshold. All subsets adhere to a protein-level fold-change threshold of 1.5. Subsets in (a, b) thus based on the n=601 and n=79 proteins, corresponding to the dark red and dark blue dots in* ***Figure 2a****, and the light and dark red and blue dots in* ***Figure 2b****, respectively. Subsets in (c, d) comprise n=1219 and n=358 proteins, respectively, equating to all the dots outside the dashed vertical fold-change threshold lines. The x-axis represents the log2 enrichment score, while the y-axis shows the transformed FDR. GO terms are represented by circles, where the size corresponds to the number of proteins linked to each GO term, visualizing the difference of significant proteins associated with the control strain (left) compared to the clinical strain (right). The circles are color-coded as light red for over-represented (enriched) and light blue for under-represented ("under-enriched") terms.* ***Table 1a*** *and* ***1b*** *provide additional information on the terms labeled in capital letters for (a) and (d) respectively. The corresponding results for (b) and (c) are presented in Supplementary Tables S5 and S6 respectively.*
